# High-speed infrared thermal measurements of impacted metallic solids

**DOI:** 10.1016/j.mex.2020.100914

**Published:** 2020-05-13

**Authors:** J.C. Nieto-Fuentes, S. Osovski, D. Rittel

**Affiliations:** a Department of Continuum Mechanics and Structural Analysis, University Carlos III of Madrid, 28911 Leganés, Madrid, Spain; b Faculty of Mechanical Engineering, Technion, 32000 Haifa, Israel

**Keywords:** Taylor-Quinney factor, Thermomechanics, High-rate deformation, Kolsky bar

## Abstract

The methodology used to measure transient temperature changes in impacted solids, using high-speed infrared detectors, is presented and discussed thoroughly. The various steps leading to a reliable measurement, namely selection of the sensing device, calibration of the setup, interfacing with the impact apparatus (Kolsky bar), and data reduction are presented. The outcome of the above methodology is illustrated in terms of the Taylor-Quinney factor, a well-known measure of the efficiency of the thermomechanical conversion.

• Selection of infrared detectors.
• Importance of the calibration procedure.
• Determination of the Taylor-Quinney factor.

## Graphical abstract

**Figure:**
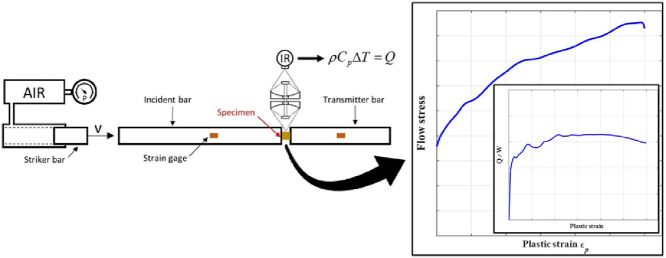

Specifications table

**Table:** 

Subject Area:	Engineering
More specific subject area:	Impact mechanics
Method name:	Quantification of the heat generated under high-speed plastic deformation
Name and reference of original method:	Moss, G.L., Pond, R.B., 1975. Inhomogeneous thermal changes in copper during plastic elongation. Metall. Trans. A 6, 1223–1235. D.O.I.: 10.1007/BF02658532
Resource availability:	N/A

## Method details

### Introduction

Since the early forging experiments of Tresca, back to 1878 [1], mechanicians are still seeking to properly characterize the thermomechanical behavior of metals under impact. In such situations – easily encountered in bird strikes on aircraft engines, ballistic impact on military armor, or vehicle crash events – a sudden increase of temperature is expected, due to the short time scale of the impact events [2]. As a result, microstructural transformations may develop in the material, leading to an apparent and sudden catastrophic failure. While one could think about possible directions to minimize these effects, quantification of the heat generated under high-speed deformation is a prerequisite. Farren and Taylor [3], and later on Taylor and Quinney [4], set a milestone on the assessment of the fraction of dissipated energy during plastic deformation, using a calorimeter. As a matter of fact, the ratio of the thermomechanical conversion (plastic work to heat) has been called since then the Taylor-Quinney factor (TQF or *β_int_*) which, assuming adiabatic conditions can be defined as [5]:

(1) $$\beta_{\text{int}}=\frac{\rho C_{p}\Delta T}{\int dW_{P}}$$

where *ρ* is the material density, *C_p_* is the heat capacity, *ΔT* is the temperature rise during deformation (the required quantity to be measured), and *W_P_* corresponds to the plastic or mechanical work (the input energy). The numerator of Eq. 1 is the energy dissipated as heat.

Three main types of detectors can be mentioned: thermal detectors, photon detectors, and photoconductive detectors [6]. Thermal detectors, e.g., thermocouples, are basically temperature-voltage transducers: a junction of two different metals that, under radiant energy absorption, will cause a change in the measured electrical response (voltage difference). The time response of this type of detectors, unfortunately, is much greater than the characteristic one for dynamic loading, making them not suitable for impact scenarios, although specific implementations of the technique have been proposed [7]. The second group, also called photodiodes, are detectors based on photon effects. When photons irradiate the semiconductor p-n junction, a voltage potential or a photo current is generated; these detectors are faster than the thermal ones, with a high performance between short to medium infrared (IR) wavelength range [8]. An example of a widely used photodiode for high temperature applications is the InSb (indium antimonide) based, that features maximum sensitivity between 1 to 5.5 µm in the electromagnetic spectrum. The third group, photoconductive detectors, do not produce a current under the effect of irradiation (unlike photodiodes). In this case, the excitation of the semiconductor results in a decrease of the junction's electrical resistance. An externally applied bias voltage closes the circuit. These detectors are as fast as photodiodes and present a good response in the medium to long infrared spectrum, making them the workhorse of dynamic experimentation at room temperature^1^. Both photodiodes and photodetectors need to operate at cryogenic temperatures to improve the spectral detectivity, otherwise the ambient existent thermal noise will impair the detector's performance. Considering specifically non-invasive measurements and taking into account that thermal full-field cameras have a response time of the order of the millisecond, while a typical impact experiment requires a temporal resolution of the order of the microsecond, the only suitable apparatus consists of infrared detectors. On the other hand, attempts have been made to increase the poor spatial resolution of IR detectors by considering one-dimensional (2 to 8 pixels) or two-dimensional (8 to 64 pixels) arrays of multiple elements. These configurations have been shown to be the substitute of thermal cameras in applications where high temporal and spatial resolution is fundamental, e.g., in dynamic fracture problems to measure the temperature evolution of a propagating crack [9]. In less arduous dynamic deformation experiments (like the one addressed in this work), where a one-dimensional state of stress and homogeneous deformation exist [10], a single detector element can be used.

In the original work of Moss and Pond in 1975 [11], a copper-doped germanium photodiode detector was used to measure the temperature rise of copper under medium-rate tensile deformation. These detectors are characterized by a wide response within the electromagnetic spectrum (2-25 µm), although the peak spectral detectivity (around 20 µm) is far from that of room temperature applications [12] (following Wien's displacement law, this corresponds to a blackbody temperature of approximately 145 K). In 1987, Hartley et al. [13] presented one of the first applications of infrared techniques combined with Kolsky bar experimentation (to be explained later on), to measure the heat dissipated during shear band formation in steels. Here, a linear array of InSb detectors were used and, due to their nature, the minimum temperature recorded was about 100°C (see Fig. 11 in [13]), thus the temperature rise at the initial stages of the plastic deformation could not be captured. Another issue the authors found in their work, was the location of the reflective mirror (used to concentrate the radiation emitted from the specimen in the focal point of the detector, as explained later on), which forced the detector to be placed out of axis with respect to the specimen.

In the work presented here, we propose an effective method to measure *in situ* infrared thermal signals during high-speed deformation of metals at room temperature. Firstly, we show the experimental setup and the specimens used. Then, an explanation of the IR calibration is followed by the procedure to synchronize the thermal and the mechanical signals. Finally, the calculation leading to the TQF from the stress-strain curve and the temperature profile is explained.

### Experimental setup

**Fig. 1:**
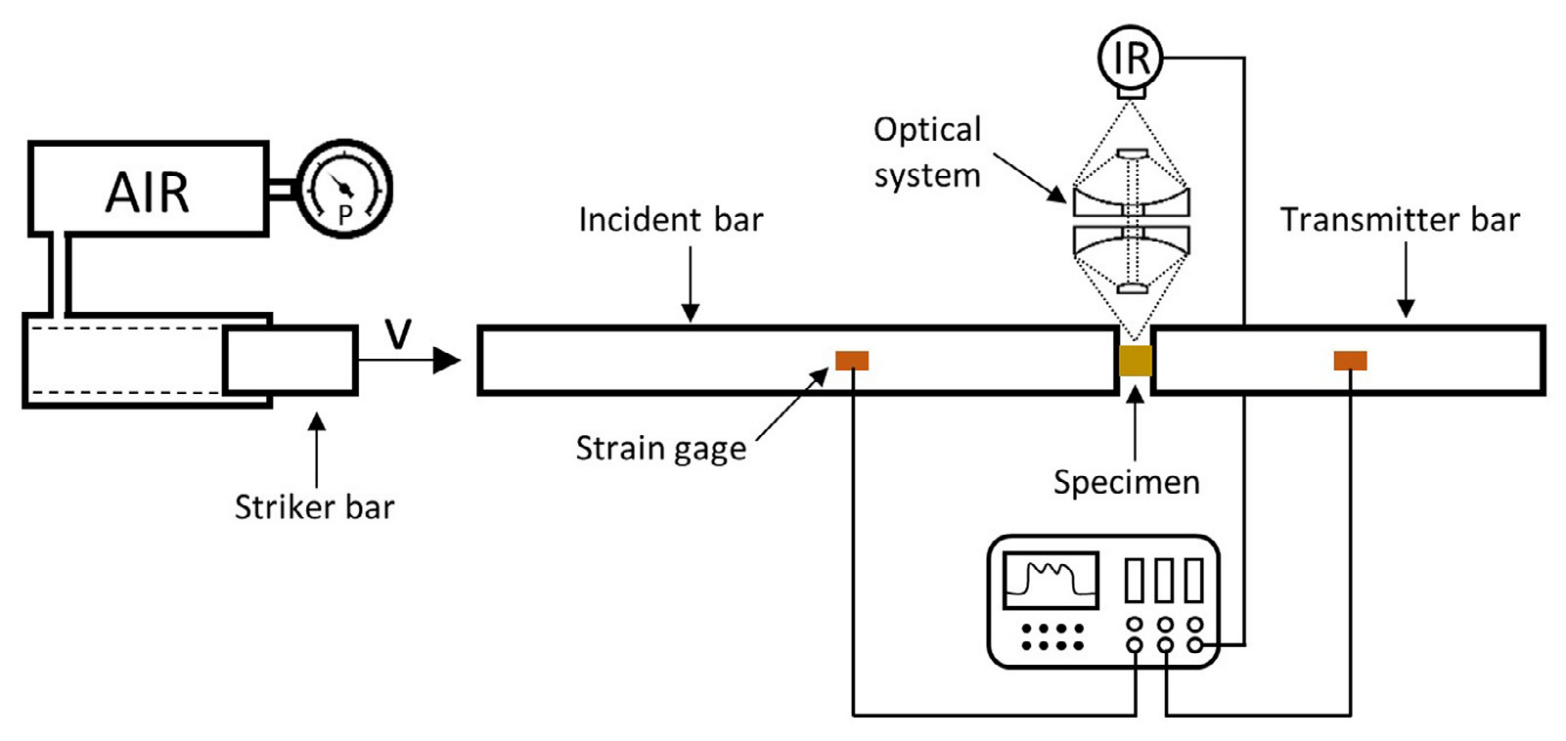
Schematic representation of the experimental setup for impact Kolsky bar tests, combined with *in situ* high-speed IR thermal measurements. See also in Fig. 2 of [17] an actual view of the setup.

**Fig. 2:**
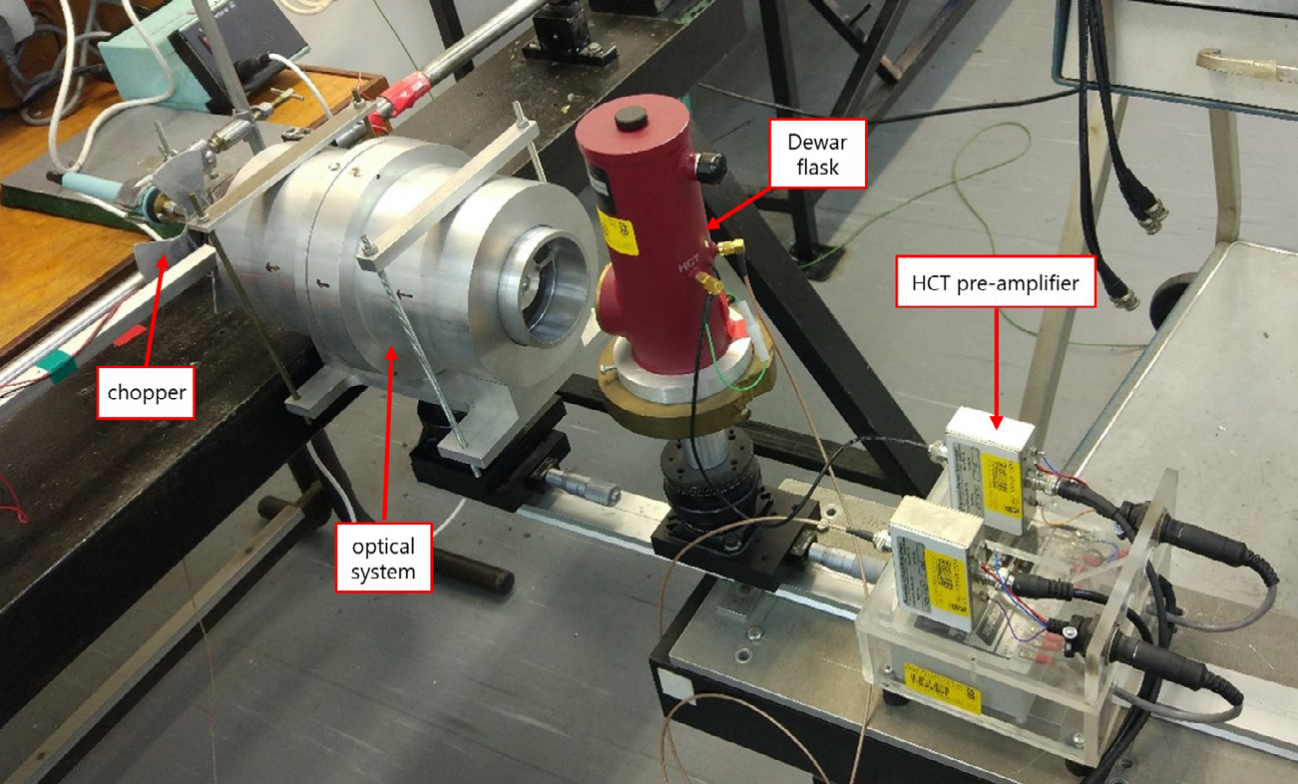
Experimental setup during IR calibration.

Dynamic experiments are conducted on a split Hopkinson pressure bar (SHPB or Kolsky bar) [14], in combination with a mercury-cadmium-telluride (HgCdTe) high-speed IR detector – see a schematic representation in Fig. 1. The Hg_1-x_Cd_x_Te compound (commonly known as MCT) is one of the most common semiconductors used in photoconductive detectors, and can be tailored to change the wavelength peak response by changing the molar fraction *x* [15]. The specific detector used in this work (and in previous works of these and other authors [16], [17], [18]) was customized by *InfraRed Associates, Inc.* with an active element area of 250 × 250 µm^2^, covering radiation emitted from the specimen within a waveband of 5.5 to 12 µm (with a peak spectral detectivity around 11 µm) in the electromagnetic spectrum, being suitable for the type of room-temperature dynamic deformation tests addressed in this work. The photoconductor is encapsulated on the top of a cold finger in a side-looking metal Dewar flask (see the maroon device in Fig. 2), cooled in liquid N_2_ down to cryogenic temperatures (77K), to prevent thermal noise. A ZnSe window, transparent to radiation from 2 to 14 µm, separates the detector from the exterior. The radiation emitted from the specimen while heating up, has to be “focused” on the detector's element surface. For that purpose, it is a good practice in optics to use mirrors rather than lenses, since the former are achromatic and do not suffer from a possible spherochromatism. Thus, a 1:1 magnification optical system was situated in-axis between the specimen and the detector's window (see a simplified drawing in Fig. 1, and the aluminum cylindrical case in Fig. 2). The configuration of the reflective optical system was presented in [19], and consists of two concave and two convex protected gold mirrors, which is basically a modification of the classical Schwarzschild design, avoiding in this case a possible shading if ones locates the specimen between the detector and the optical system (as in [13]). Moreover, a specimen subjected to large deformations under axial compression experiences radial expansion, creating a motion of the “focused” surface towards the detector. This is not a major concern in radiometric techniques, like the one addressed in this work, since the *focus* here is the origin of radiation emission and heat flux should not be affected by the motion of the surface (unlike in imaging systems, where a loss of focus can blur the image) – see [20].

**Fig. 3:**
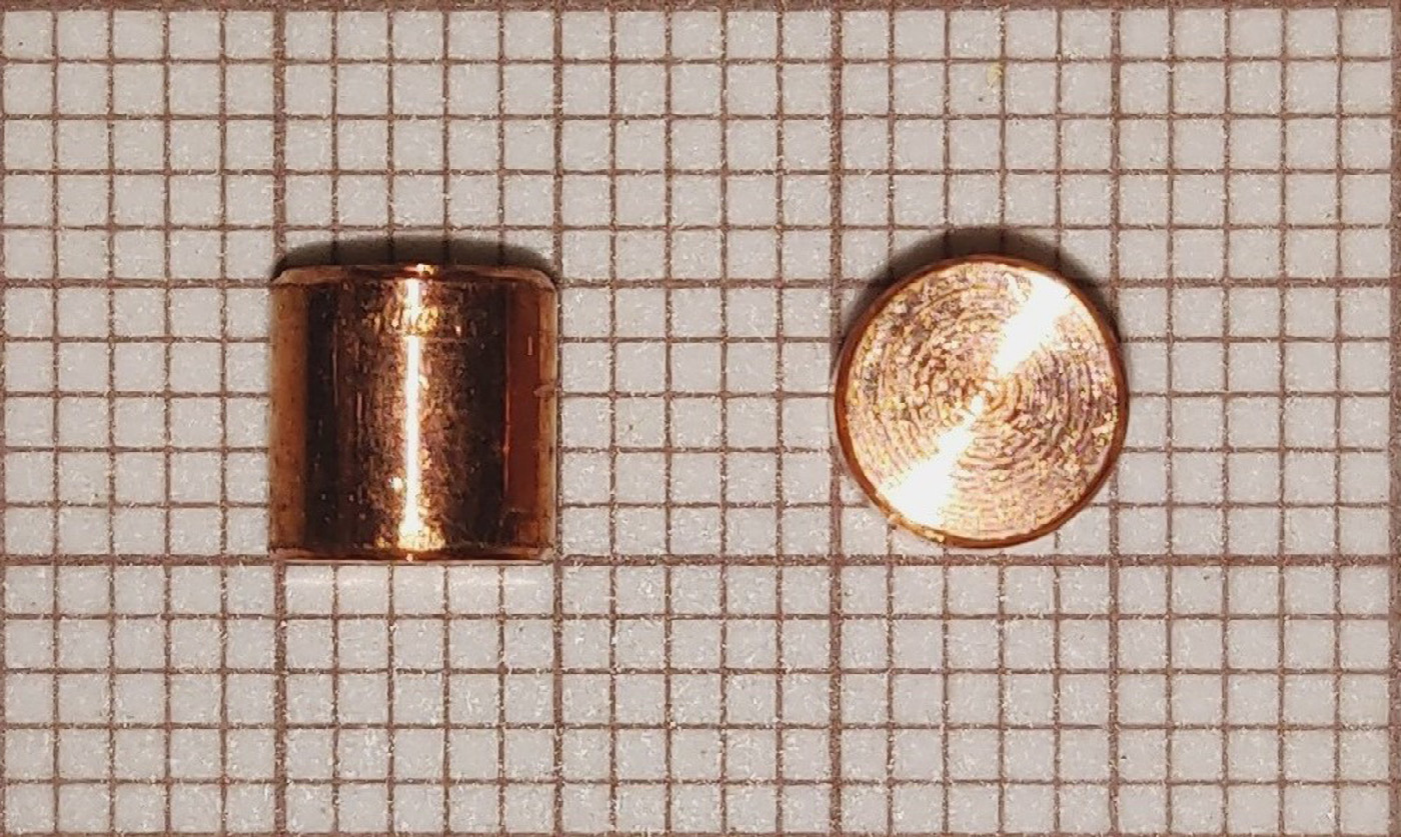
Pure Cu 5 × 5 mm cylindrical specimens over millimeter paper on the background.

The signals from the strain gages in both incident and transmitter bar, in addition to the one of the IR detector, have to be recorded simultaneously. As will be later explained, the signals have to be synchronized to calculate correctly the thermomechanical conversion ratio. The acquisition frequency of our system allows to record data every 0.5 µs – fast enough to capture the events under Kolsky bar experimentation. The type of specimens considered throughout this work were compression cylinders, which can vary in length and diameter to control the applied strain rate – see in Fig. 3 an example of machined copper cylinders. On the other hand, the IR thermal measurement technique presented here is not limited to compression tests, and can be applied to other SHPB configurations, such as tension or torsion loading setups.

### IR calibration

Prior to each test, it is important (if not mandatory) to conduct a thermal calibration on the IR detector, i.e., a relation between the voltage signal output from the latter and the temperature emitted from the specimen has to be established. While it would be highly desirable to relate the emissivity of the sample material and the amount of radiation emitted from the specimen to determine the temperature rise, these parameters are difficult to measure, thus a direct relationship between voltage and temperature is preferred [9]. Depending on the material, surface conditions may change during deformation, although a minor dependence of the calibration curve (emissivity) on the specimen's surface roughness is expected [21], [22], [23].

**Fig. 4:**
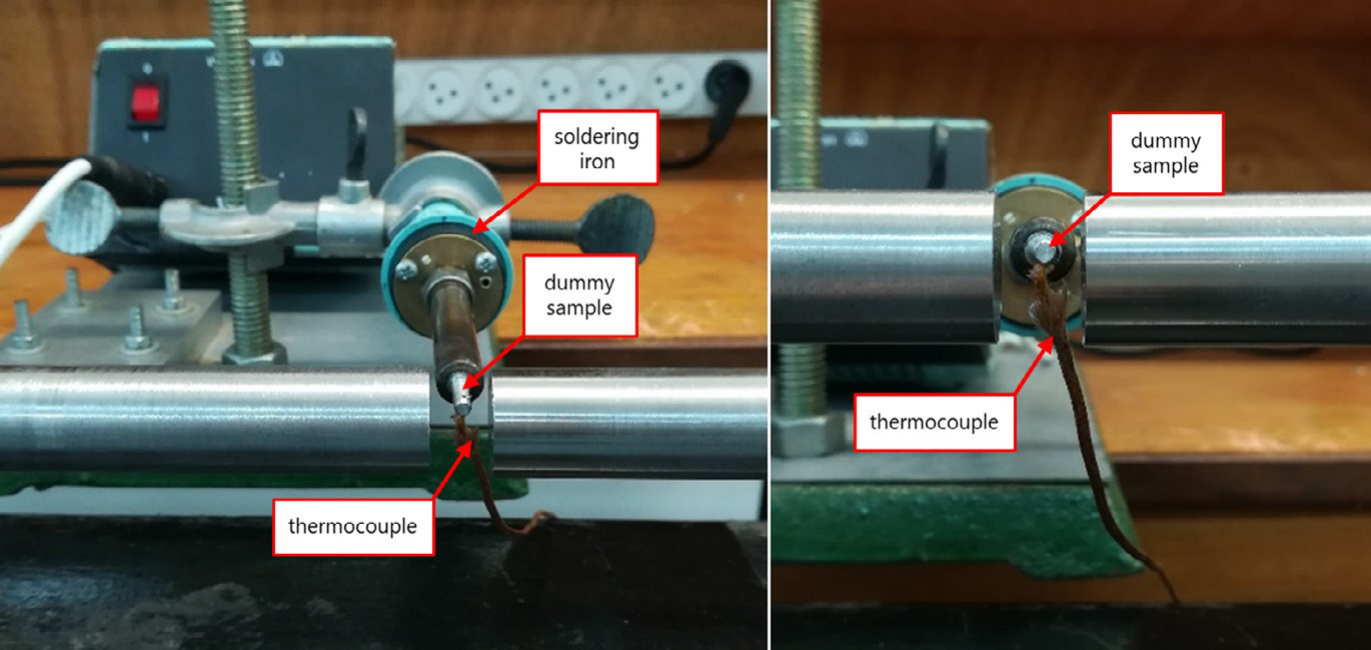
Dummy specimen (here, pure aluminum) situated on the tip of a soldering iron. The K-type thermocouple is coiled around the sample.

**Fig. 5:**
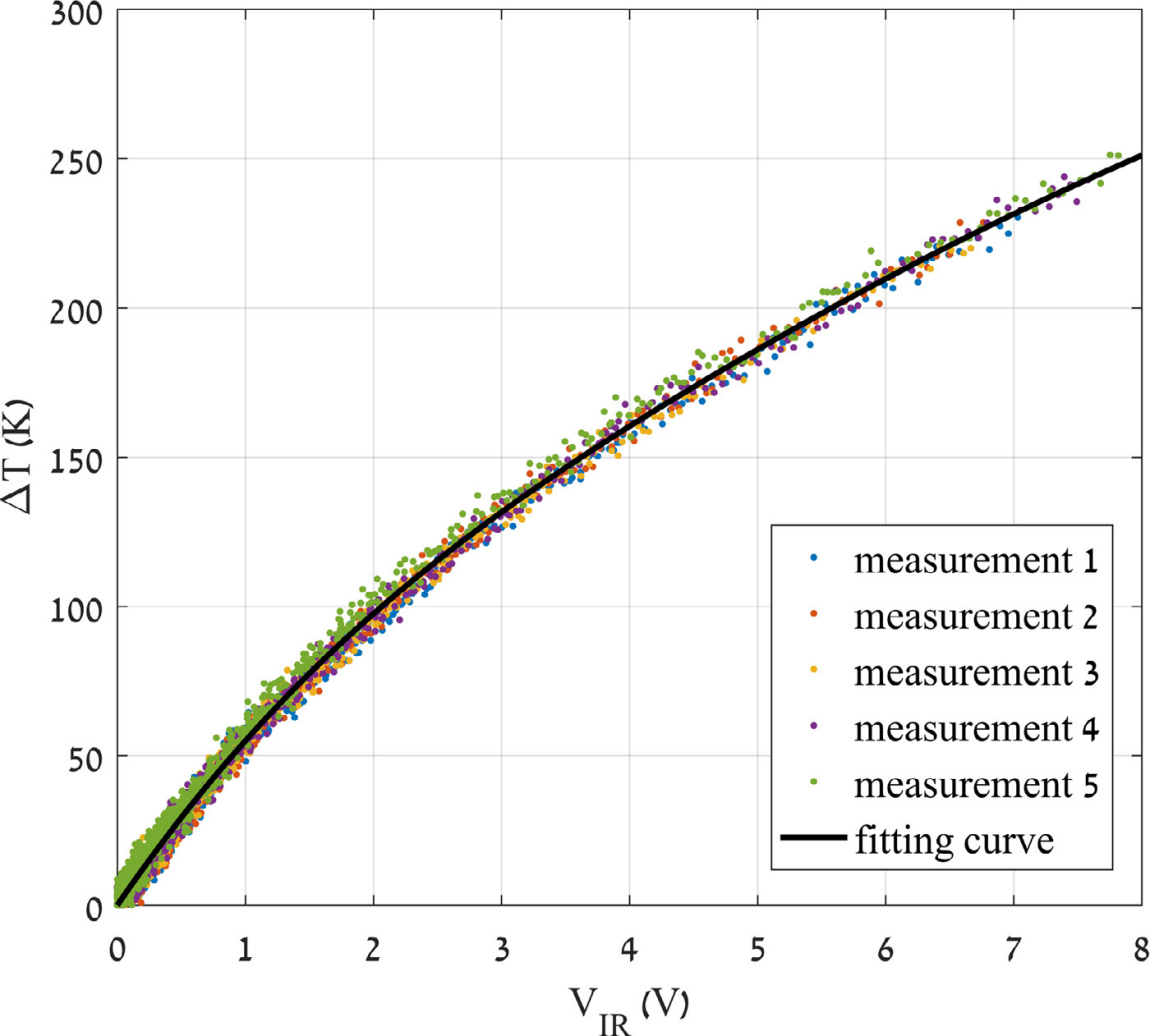
Increment of temperature (K) *vs.* IR detector signal (V).

For that purpose, a dummy sample of the same material to be tested (ideally with the same surface finishing) has to be placed at the tip of a soldering iron – see Fig. 4 – in the same location as the actual specimen will be. A small hole, drilled in the dummy specimen, will host a K-type thermocouple. The following procedure is to heat up the dummy specimen and adjust the position of the bars/specimen/detector to find the focal point of the detector. A piece of cardboard with a hole of the size of the specimen will help to find an accurate locus. Note here that during the signal recording, the radiant energy has to be modulated in time, otherwise the photoconductor signal will remain constant with minimum responsivity [15]. In our case, a home-made optical chopper is located between the heat source and the detector (see a graphical example in Fig. 2); one should adjust the angular speed of the chopper until the responsivity of the detector is at (or near) its maximum. Now, once the thermal signal shows a maximum peak-to-valley response^2^, the heated dummy sample is let to cool down to room temperature, recording the signal of both the IR detector and the K-type thermocouple. Since the relation between the thermocouple voltage output and the actual temperature is known, that of the measured by the photoconductor can be obtained. This procedure must be repeated several times, to ensure a good repeatability before obtaining a voltage-temperature fitting curve. If the material has never been tested before, it is a good practice to record at least four sets of calibration points (i.e., repeating the cycle of heating the dummy specimen and recording the data while cooling it down) before obtaining the fitting curve. If the material is already known, we recommend collecting two sets of points and verify that the curve falls into the expected values. Note that even different atmospheric conditions in the laboratory (in terms of moisture) may play an important role on infrared wave attenuation [24] In Fig. 5 we show an example of a calibration curve for pure nickel, adjusted using the *Curve Fitting* tool provided by MATLAB^Ⓡ^.

### Experimental procedure and signal synchronization

Once the thermal calibration is accomplished, the soldering iron and the dummy specimen can be removed from the setup. Likewise, the optical chopper is no longer necessary hereinafter, since the temperature gradients under the dynamic tests produce a transient response of the infrared photoconductive detectors.

**Fig. 6:**
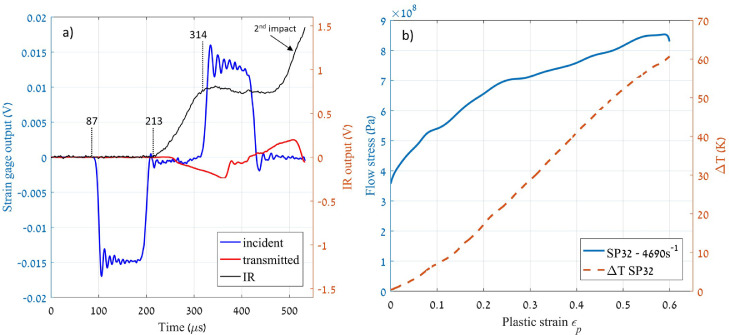
*a)* Recorded signals during the dynamic tests. *b)* Flow stress (Pa) and temperature rise (K) *vs.* plastic strain of a pure nickel specimen deformed at 4690 s^−1^.

In a regular Kolsky bar test, three types of signals are obtained: incident and transmitter strain gages, and the temperature rise – see the corresponding raw signals in Fig. 6a. A synchronization of the signals must be performed since, while the thermal ones are measured directly on the specimen, the mechanical characteristics are usually measured using strain gages located away from the specimen. Consequently, the elastic wave signals must be shifted (considering the distances between strain gages and the specimen) according to the wave velocity in the bars for them to correspond with the thermal ones, allowing for a fully synchronized record of the stress-strain-temperature characteristics. Following the notes in Fig. 6a:

• after the impact of the striker, the generated stress wave passes through the incident strain gage at time t_1_ = 87 µs;
• from the latter point, we consider the time that the stress wave devotes on travelling the distance between the incident strain gage and the specimen, in addition to the time that the specimen is deforming elastically, i.e., t_2_ = 213 µs;
• finally, we take that part of the temperature (IR) signal corresponding to the stage of plastic deformation, until t_3_ = 314 µs.

It is a good practice in this type of experiments to verify if the time shift was performed accordingly. For that purpose, one can represent in the same figure the stress vs. plastic strain together with the temperature profile. A good indication of the success of the experiment is that the temperature rise should start around zero plastic strain, up to the failure (or the end of the deformation) of the specimen – see an example in Fig. 6b. As a remark, note that the specimen may suffer from multiple impacts during the test, as one can clearly see in the second stage of the temperature rise in Fig. 6a. In our work, the first impact is only considered.

### Calculation of the Taylor-Quinney factor

**Fig. 7:**
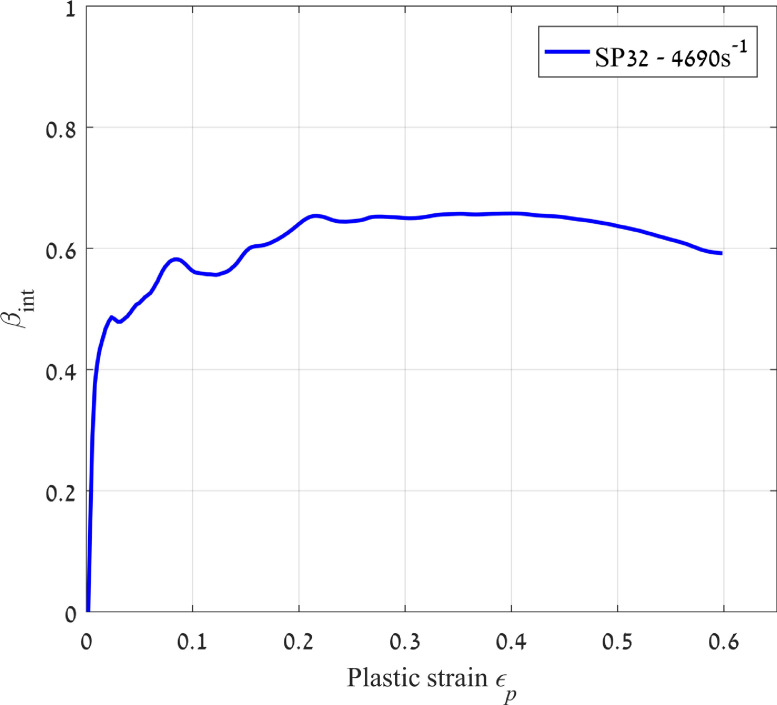
Taylor-Quinney factor vs. plastic strain of a pure nickel specimen deformed at 4690 s^−1^.

The main objective of the *in situ* thermal measurements is the calculation of the TQF (see Eq. 1), which gives at the same time, an idea of the amount of energy that remains stored in the material after deformation. Here, following Eq. 1, the increment of temperature is multiplied by the density and the heat capacity of the material, to be then divided by the area under the stress-strain curve. The result is the curve presented in Fig. 7.

## Method validation

Recent works where the previous methodology was successfully proven can be found in [17,18].

## Declaration of Competing Interest

The authors declare that they have no known competing financial interests or personal relationships that could have appeared to influence the work reported in this paper.

## Footnotes

1 For a body radiating from room temperature and above, the peak responsivity falls within a wavelength of the propagating waves in the long to mid-infrared of the electromagnetic spectrum.

2 With the system used here: ±10V after signal amplification, for a temperature of approximately 300°C.

## References

[1] Tresca M.H. (1878). Proc. Inst. Mech. Eng..

[2] Boley B.A., Weiner J.H. (1960). Theory of Thermal Stresses.

[3] Farren W.S., Taylor G.I. (1925). Proc. R. Soc. A Math. Phys. Eng. Sci..

[4] Taylor G.I., Quinney H. (1934). Proc. R. Soc. A Math. Phys. Eng. Sci..

[5] Rittel D. (1999). Mech. Mater..

[6] Riedl M.J. (2001). Optical Design Fundamentals for Infrared Systems.

[7] Rabin Y., Rittel D. (1999). Exp. Mech..

[8] Schlessinger M. (1994). Infrared Technology Fundamentals.

[9] Zehnder A.T., Rosakis A.J. (1991). J. Mech. Phys. Solids.

[10] Ravichandran G., Subhash G. (1994). J. Am. Ceram. Soc..

[11] Moss G.L., Pond R.B. (1975). Metall. Trans. A.

[12] Wolfe W.L., Zissis G.J. (1978). The Infrared Handbook.

[13] Hartley K.A., Duffy J., Hawley R.H. (1987). J. Mech. Phys. Solids.

[14] Kolsky H. (1949). Proc. Phys. Soc. Sect. B.

[15] Daniels A. (2010). Field Guide to Infrared Systems, Detectors, and FPAs.

[16] Zhang L.H., Rittel D., Osovski S. (2018). Mater. Sci. Eng. A.

[17] Nieto-Fuentes J.C., Rittel D., Osovski S. (2018). Int. J. Plast..

[18] Nieto-Fuentes J.C., Osovski S., Venkert A., Rittel D. (2019). Phys. Rev. Lett..

[19] Rittel D., Wang Z.G. (2008). Mech. Mater..

[20] Regev A., Rittel D. (2008). Exp. Mech..

[21] Hodowany J., Ravichandran G., Rosakis A.J., Rosakis P. (2000). Exp. Mech..

[22] Mason J.J., Rosakis A.J., Ravichandran G. (1994). Mech. Mater..

[23] Rittel D., Bhattacharyya A., Poon B., Zhao J., Ravichandran G. (2007). Mater. Sci. Eng. A.

[24] Elder T., Strong J. (1953). J. Franklin Inst..

